# The Regulatory Small RNA MarS Supports Virulence of *Streptococcus pyogenes*

**DOI:** 10.1038/s41598-017-12507-z

**Published:** 2017-09-25

**Authors:** Roberto Pappesch, Philipp Warnke, Stefan Mikkat, Jana Normann, Aleksandra Wisniewska-Kucper, Franziska Huschka, Maja Wittmann, Afsaneh Khani, Oliver Schwengers, Sonja Oehmcke-Hecht, Torsten Hain, Bernd Kreikemeyer, Nadja Patenge

**Affiliations:** 1 Institute of Medical Microbiology, Virology and Hygiene, University Medicine Rostock, Rostock, Germany; 2 Core Facility Proteome Analysis, University Medicine Rostock, Rostock, Germany; 3 Institute for Medical Microbiology, Justus-Liebig University of Giessen, Giessen, Germany; 4 Institute for Medical Microbiology, Justus-Liebig University of Giessen, Giessen, Germany; 5 Present Address: Franziska Huschka, Institute for Medical Microbiology, Virology and Hygiene, University Medical Center Hamburg-Eppendorf, Hamburg, Germany

## Abstract

Small regulatory RNAs (sRNAs) play a role in the control of bacterial virulence gene expression. In this study, we investigated an sRNA that was identified in *Streptococcus pyogenes* (group A *Streptococcus*, GAS) but is conserved throughout various streptococci. In a deletion strain, expression of *mga*, the gene encoding the multiple virulence gene regulator, was reduced. Accordingly, transcript and proteome analyses revealed decreased expression of several Mga-activated genes. Therefore, and because the sRNA was shown to interact with the 5′ UTR of the *mga* transcript in a gel-shift assay, we designated it MarS for *m* *ga*-activating regulatory sRNA. Down-regulation of important virulence factors, including the antiphagocytic M-protein, led to increased susceptibility of the deletion strain to phagocytosis and reduced adherence to human keratinocytes. In a mouse infection model, the *marS* deletion mutant showed reduced dissemination to the liver, kidney, and spleen. Additionally, deletion of *marS* led to increased tolerance towards oxidative stress. Our *in vitro* and *in vivo* results indicate a modulating effect of MarS on virulence gene expression and on the pathogenic potential of GAS.

## Introduction

*Streptococcus pyogenes* (group A *Streptococcus*, GAS) is a strictly human pathogen that is responsible for a variety of infections of distinct severity^1^. While superficial infections of the upper respiratory tract and the skin can be treated effectively with antibiotics, invasive streptococcal diseases remain life-threatening. The development of invasive infections and immune sequelae involves at least one or more of the following parameters: insufficient treatment of a primary infection, persistence of streptococci in the host tissue, and the expression of specific virulence factor genes by the bacteria^2^. Control of virulence gene expression by stand-alone transcription factors and two-component systems are known to play a role in virulence determination in GAS^3^. An additional level of bacterial gene expression control is provided by small regulatory RNAs (sRNAs)^4^. Bacterial sRNAs can serve as negative regulators by inhibiting translation or by decreasing mRNA stability and can also function as positive regulators by stabilizing mRNA transcripts or initiating the translation of mRNAs^5,6^. Although several global sRNA screens have recently been performed in streptococci, the RNA-dependent regulatory network in GAS is not yet well understood^7^.

Several *trans*-acting sRNAs, targeting mRNA sequences by direct base pairing, have been discovered in GAS. The pleiotropic effect locus (Pel) was described to have an effect on virulence and to influence the production of several virulence factors, including the M-protein, the cysteine protease SpeB, fibronectin-binding protein, and streptokinase^8^. The untranslated mRNA of *pel*, which also contains the gene for the streptolysin S peptide (*sagA*), was shown to be an effector of virulence factor expression in GAS^9^. However, in the GAS M1T1 lineage, no regulatory function of PEL could be observed^10,11^.

The sRNA gene *rivX* is located downstream of the transcriptional regulator gene *rivR*. While RivR was shown to affect transcriptional activation by Mga, possibly by interacting with Mga protein, RivX was hypothesized to act through a separate but as yet unknown pathway to increase the expression of multiple genes that are regulated directly or indirectly by Mga^12^. In a conflicting report, RivR was shown to be a negative regulator of capsule production in GAS while no regulatory function could be assigned to RivX. No influence of the *riv*R/X locus on the expression of *mga* or *mga*-associated genes was detected in this study^13^.

Until now, the only extensively characterized and functionally validated sRNA in GAS was FasX. FasX acts as positive regulator of the *fasBCA* operon, coding for the fibronectin/fibrinogen binding/haemolytic activity/streptokinase regulator^14^. FasX was shown to positively affect streptokinase production by stabilizing *ska* mRNA^15^. FasX also acts as a negative regulator of pilus expression by destabilizing the pilus operon mRNA and inhibiting the translation of the *cpa* transcript, which encodes a minor pilus protein^16^. By binding to and inhibiting the translation of different mRNAs of the fibronectin, collagen, T-antigen (FCT) region, control over the pilus gene region by FasX varies in a serotype-specific fashion^17^. In a subsequent report, FasX was shown to negatively control the production of two fibronectin-binding proteins, PrtF1 and PrtF2, encoded by the FCT region^18^.

In this study, we phenotypically characterize the GAS wild-type strain 591 (referred to as GAS M49 591 throughout the text) lacking the *m* *ga*-activating regulatory sRNA (MarS). Employing transcript analyses, proteomics, and a mouse infection model, we show that MarS modulates Mga-dependent virulence factor gene expression, affects capsule production, and influences the fate of GAS in the host. We also show a direct interaction of MarS with the 5′ UTR of the *mga* transcript using an electrophoretic mobility shift assay (EMSA).

## Results

### Deletion of *marS* in GAS 591

**Figure 1:**
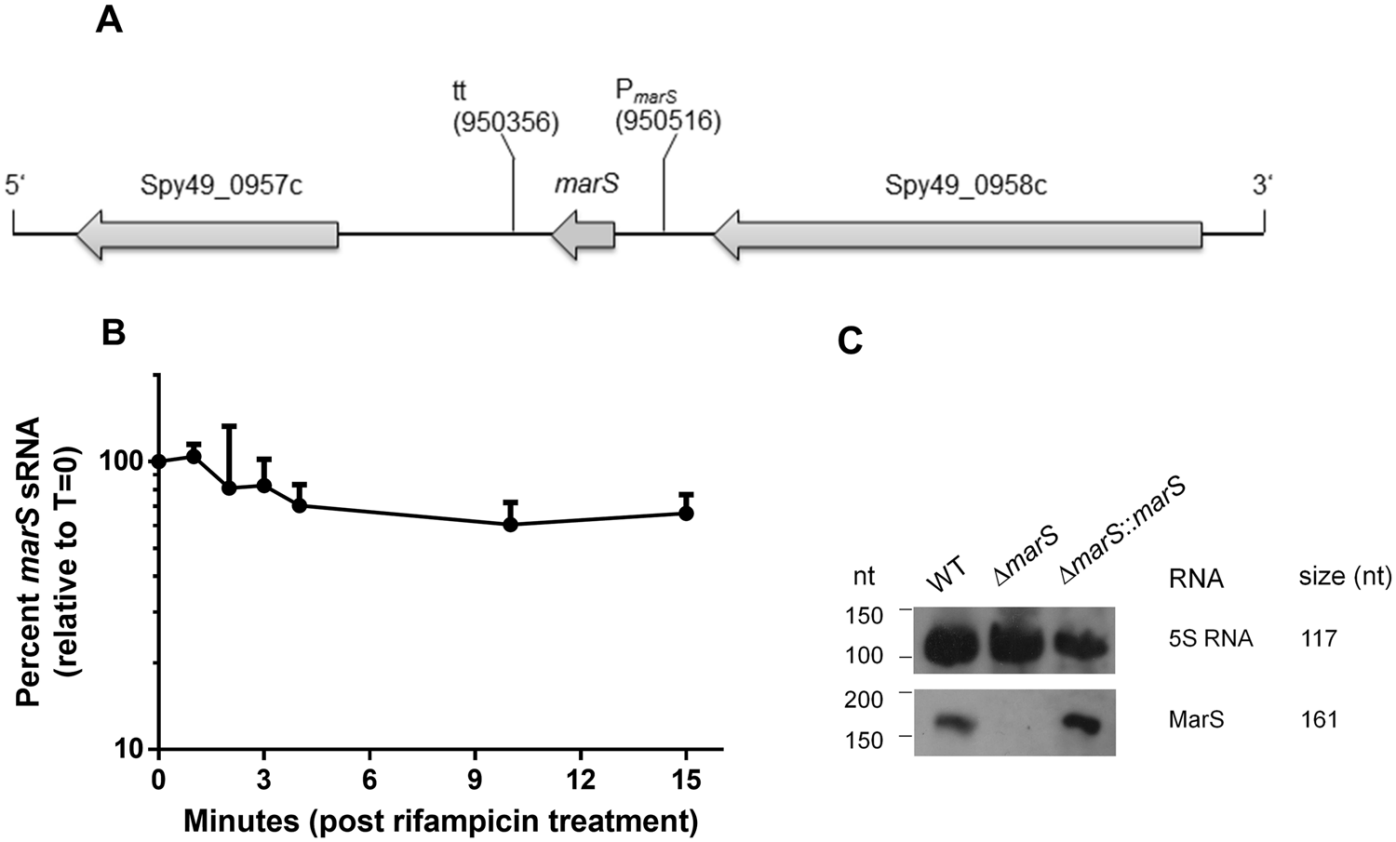
Genomic localization and transcript stability of *marS*. (**A**) Schematic diagram of the genomic locus of *marS*. Genes are represented by arrows pointing in the direction of transcription. P: first nucleotide of *marS*, tt: last nucleotide of *marS*. (**B**) Stability of MarS in GAS M49 591, determined by RT-qPCR following treatment of the culture with rifampicin. The data are presented as the percent *marS* transcript levels relative to time-point zero. The mean value of three experiments ± standard deviation is shown. (**C**) Northern blot analyses of *marS* expression during growth in THY medium (OD_600_ of 0.8). The probes were specific for the RNAs indicated on the right of each blot. For comparison, the approximate sizes of the RNA, as determined by 5′ RACE analysis, are indicated on the far right. The full-length blot is presented in Supplementary Figure 3.

Genome-wide screening of the GAS wild-type strain 591 (referred to as GAS M49 591 throughout the text) resulted in an extensive list of candidate sRNA genes^19^. To identify sRNAs that were potentially involved in pathogenicity of GAS, we focused on sRNA genes that were differentially expressed in different media or throughout growth. Among those, *marS* (formerly designated sRNASpy490957c) is conserved throughout lactic acid bacteria, and a *cis*-regulatory function was predicted by a comparative genomics-based study using CMfinder^20^. However, the sRNA is transcribed independently from adjacent genes in several GAS serotypes^10,19,21^. Furthermore, in *Streptococcus mutans* and *S. pyogenes*, the presence of a terminator sequence and a strong promoter downstream of *marS* indicates that transcription is terminated prior to the downstream gene, Spy49_0957c, which is transcribed from an independent promoter^19,22^. A schematic representation of the genomic locus is presented in Fig. 1A. The sRNA gene does not encode a peptide. However, on the antisense sequence of the *marS* gene, a 108-bp ORF is located, which could lead to the expression of a 35 AA peptide for which no homologues were found by BLAST^23^ analysis. The stability of the *marS* transcript, MarS, was determined following rifampicin treatment by RT-qPCR. Transcript stability was high in GAS M49 591 (Fig. 1B), consistent with prior findings obtained in the GAS M1T1 strain MGAS2221^10^. To study the role of MarS, an isogenic deletion strain was constructed and designated Δ*marS*. Complementation of the gene deletion was achieved by ectopic expression of the sRNA gene from a shuttle vector under the control of its own promoter (Δ*marS*:*:marS*). This strategy helps exclude an influence of spurious mutations introduced during mutagenesis and was recently suggested by Cho^24^. Furthermore, whole genome sequencing was performed to rule out the occurrence of spontaneous mutations in the recombinant strains. Expression of *marS* in the respective strains was determined by Northern blot analyses of total RNA isolated from bacteria grown to the exponential phase (Fig. 1C). There was no detectable expression of *marS* in the deletion strain. By contrast, Δ*marS*:*:marS* showed increased expression in comparison to the parental strain, likely due to a higher copy number of the expression vector. These results were verified by RT-qPCR. No PCR product was detected in the RNA sample from Δ*marS*, whereas the transcript level in Δ*marS::marS* was increased 2-fold in comparison to WT. GAS M49 591 and the recombinant derivatives had similar growth characteristics in THY medium (WT: µ = 1.11 ± 0.09; ΔmarS: µ = 1.09 ± 0.09; *ΔmarS::marS*: µ = 0.89 ± 0.12). The differences between the strains were not significant (t-test). These data were generated from three independent experiments.

### Lack of MarS results in decreased survival of GAS M49 591 in human blood

**Figure 2:**
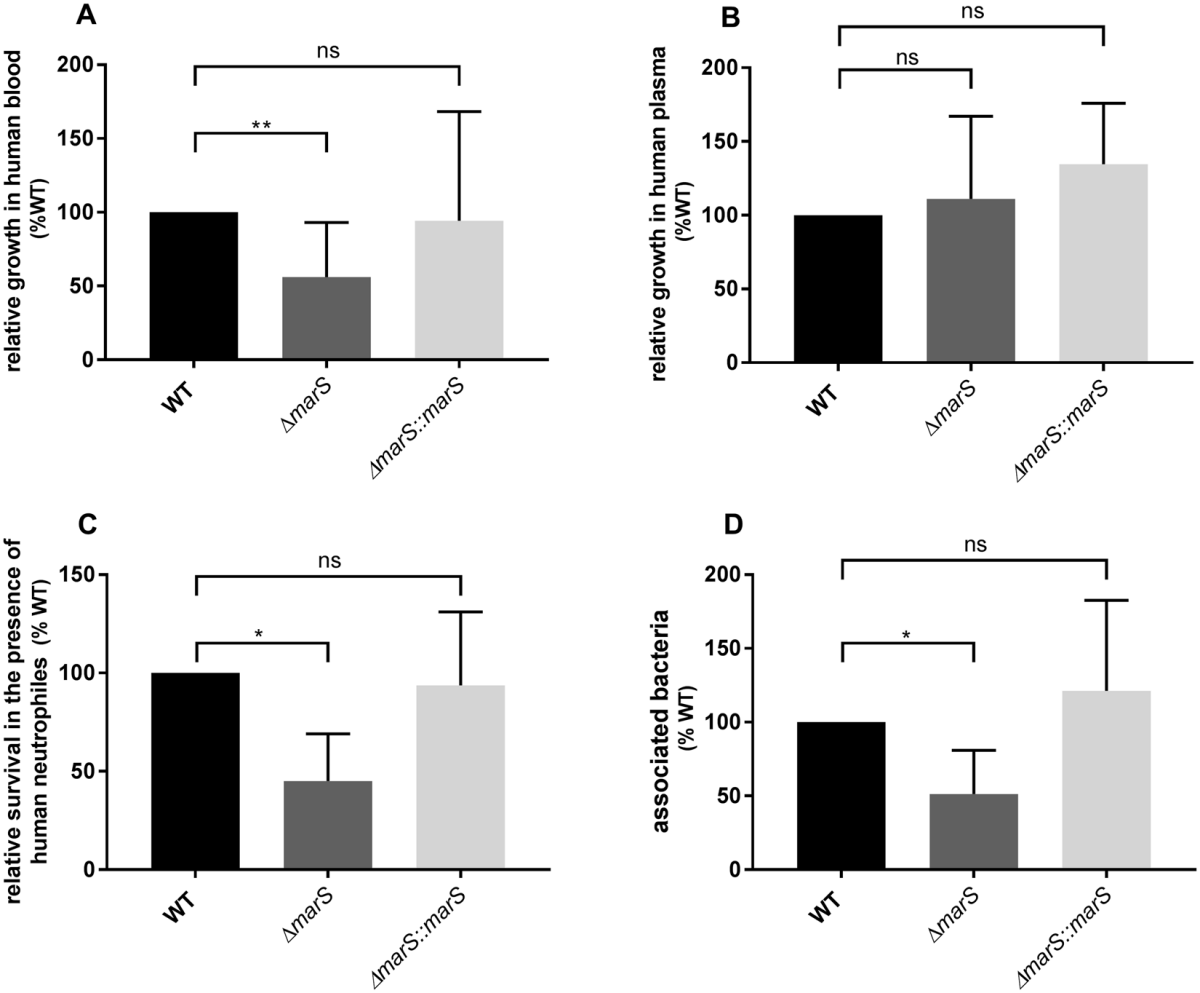
Deletion of *marS* leads to a decreased survival of GAS M49 591 in human blood and greater susceptibility to phagocytosis. (**A**) Relative growth of ∆*marS* (dark grey) and Δ*marS*::*marS* (light grey) in comparison to WT (black) in human blood, n = 12, and (**B**) in human plasma, n = 4. (**C**) Relative survival of ∆*marS* (dark grey) and Δ*marS*::*marS* (light grey) in comparison to WT (black) after incubation for 30 min with human neutrophils, n = 5. (**D**) Relative abundance of associated ∆*marS* (dark grey) and Δ*marS*::*marS* (light grey) bacteria in comparison to WT (black) after incubation with neutrophils n = 5. The Data are presented relative to WT (% WT, mean values ± standard deviation). Statistical significance was determined using the Wilcoxon signed-rank test. Differences between samples were expressed as “ns = not significant” (P ≥ 0.05), marginally significant (P < 0.05)*, and significant (P < 0.01)**.

To investigate the role of MarS in a more pathogenically relevant environment, we assessed the ability of GAS M49 591 WT, the *marS* deletion mutant, and the *marS* complementation strain to grow *ex*-vivo in human blood. ∆*marS* showed decreased growth in human blood in comparison to WT (Fig. 2A, Fig. S1). In the complementation strain, growth was restored to the WT level. To distinguish whether cellular or non-cellular components of human blood were responsible for the reduced growth of ∆*marS*, growth in human plasma was assessed. As shown in Fig. 2B and Fig. S2A, growth of ∆*marS* was not inhibited in human plasma. Consistently, the deletion of *marS* resulted in a higher susceptibility towards phagocytosis by human neutrophils (Fig. 2C, Fig. S2B). The number of extracellular bacteria was slightly reduced in the *marS* deletion strain relative to WT (Fig. 2D). Statistical analyses of non-normalized data showed that the decrease of associated bacteria was not significant (Fig. S2C).

### MarS positively influences capsule production

**Figure 3:**
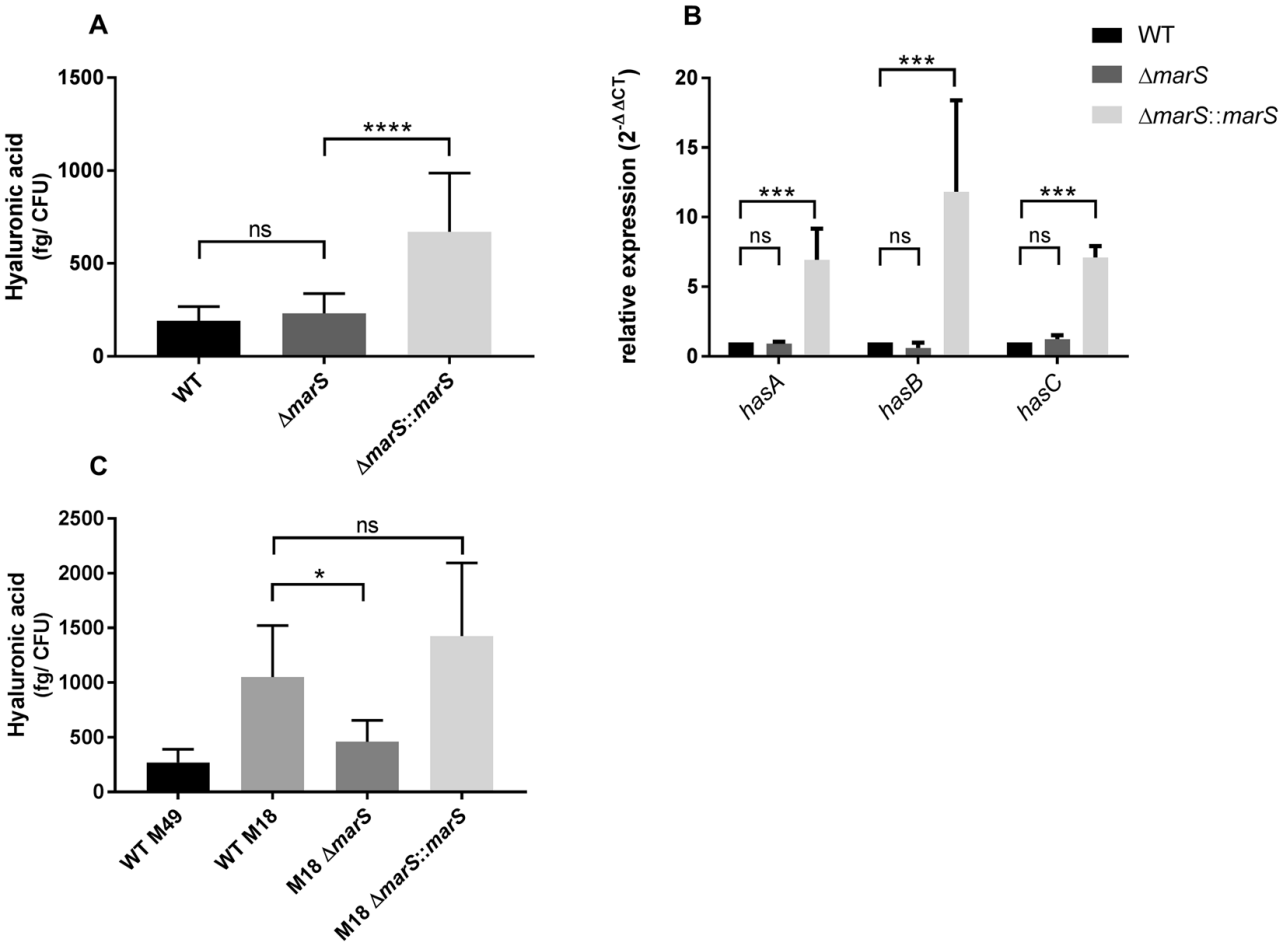
MarS positively influences capsule production by GAS. (**A**) Amount of capsule in ∆*marS* (dark grey) and Δ*marS*::*marS* (light grey) in comparison to WT (black), n = 16. (**B**) Relative expression of *hasABC*, encoding hyaluronic acid synthesis proteins, in GAS M49 591, n = 8. Statistical significance for (**B**) was determined using the two-way ANOVA, multiple comparisons. Differences between samples were expressed as “ns = not significant” (P ≥ 0.05) and highly significant (P < 0.0001)***. (**C**) Amount of capsule in GAS M49 591 (WT M49, black), GAS M18 MGAS8232 ∆*marS* (dark grey) and GAS M18 MGAS8232 Δ*marS*::*marS* (light grey) in comparison to GAS M18 MGAS8232 WT (WT M18, grey), n = 5. The data are presented as the mean values ± standard deviation. Statistical significance for (A) and (C) was determined using the two-tailed Mann-Whitney U test. Differences between samples were expressed as “ns = not significant” (P ≥ 0.05), marginally significant (P < 0.05)*, significant (P < 0.01)**, and highly significant (P < 0.0001)****.

The hyaluronic acid capsule of GAS supports evasion of the host immune system through molecular mimicry and confers resistance to phagocytosis^25–27^. Since ∆*marS* showed decreased growth in human blood and a higher susceptibility towards human neutrophils, we tested whether capsule production is impaired in this strain. No significant difference in hyaluronic acid abundance was detected between ∆*marS* and the parental strain (Fig. 3A). Interestingly, the complementation strain showed increased hyaluronic acid content in comparison to WT. This observation is in accordance with the higher *marS* transcript abundance in Δ*marS*:*:marS* (Fig. 1C) and suggests a positive effect of MarS on capsule production. To analyse a potential influence of MarS on *hasABC* mRNA abundance, we measured *hasABC* transcript abundance by RT-qPCR (Fig. 3B). The gene products of the *hasABC* operon are responsible for hyaluronic acid synthesis in GAS^28,29^. ∆*marS* showed no significant differences in *hasABC* transcript level in comparison to the parental strain, whereas the complementation strain showed a significantly increased abundance of the *hasAB*C transcripts in comparison to both WT and ∆*marS*. GAS M49 591 is characterized by a low endogenous level of capsule production^28^. Thus, the effect of MarS on hyaluronic acid synthesis was investigated in MGAS8232 (serotype M18) (referred to as GAS M18 MGAS8232 throughout the text). The hyaluronic acid production of GAS M18 MGAS8232 was considerably higher than that of M49 (Fig. 3C). The GAS M18 MGAS8232 *marS* deletion strain showed a reduced capsule production in comparison to both the GAS M18 MGAS8232 WT and the complementation strain GAS M18 MGAS8232 Δ*marS*:*:marS* (Fig. 3C). This result indicates a positive regulatory effect of MarS on capsule production in GAS.

### MarS influences adherence to human keratinocytes

**Figure 4:**
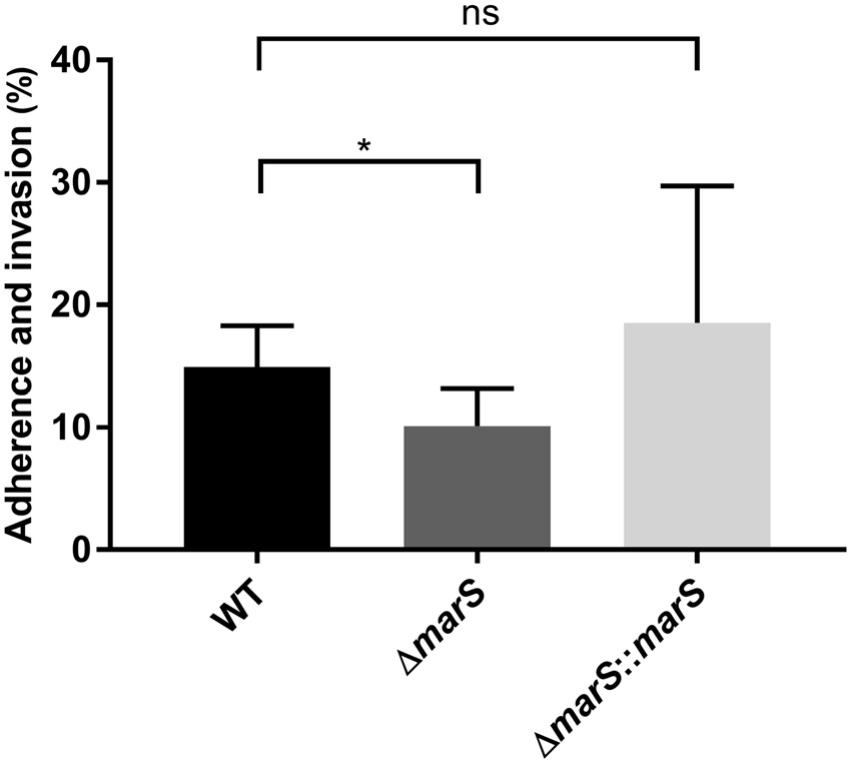
MarS influences adherence and internalization. Adherence to and internalization into human keratinocytes of ∆*marS* (dark grey) and Δ*marS*::*marS* (light grey) in comparison to WT (black), n = 6. The Data are presented as the mean values ± standard deviation. Statistical significance was determined using the two-tailed Mann-Whitney U test. Differences between samples were expressed as “ns = not significant” (P ≥ 0.05) and marginally significant (P < 0.05)*.

To test whether MarS influences adherence and invasion, bacteria were incubated with a human keratinocyte cell line (HaCaT). Deletion of *marS* led to a reduced ability to adhere to HaCaT cells, which was restored in the complementation strain (Fig. 4).

### Putative target mRNAs of MarS include *mga* and *hasB*

**Figure 5:**
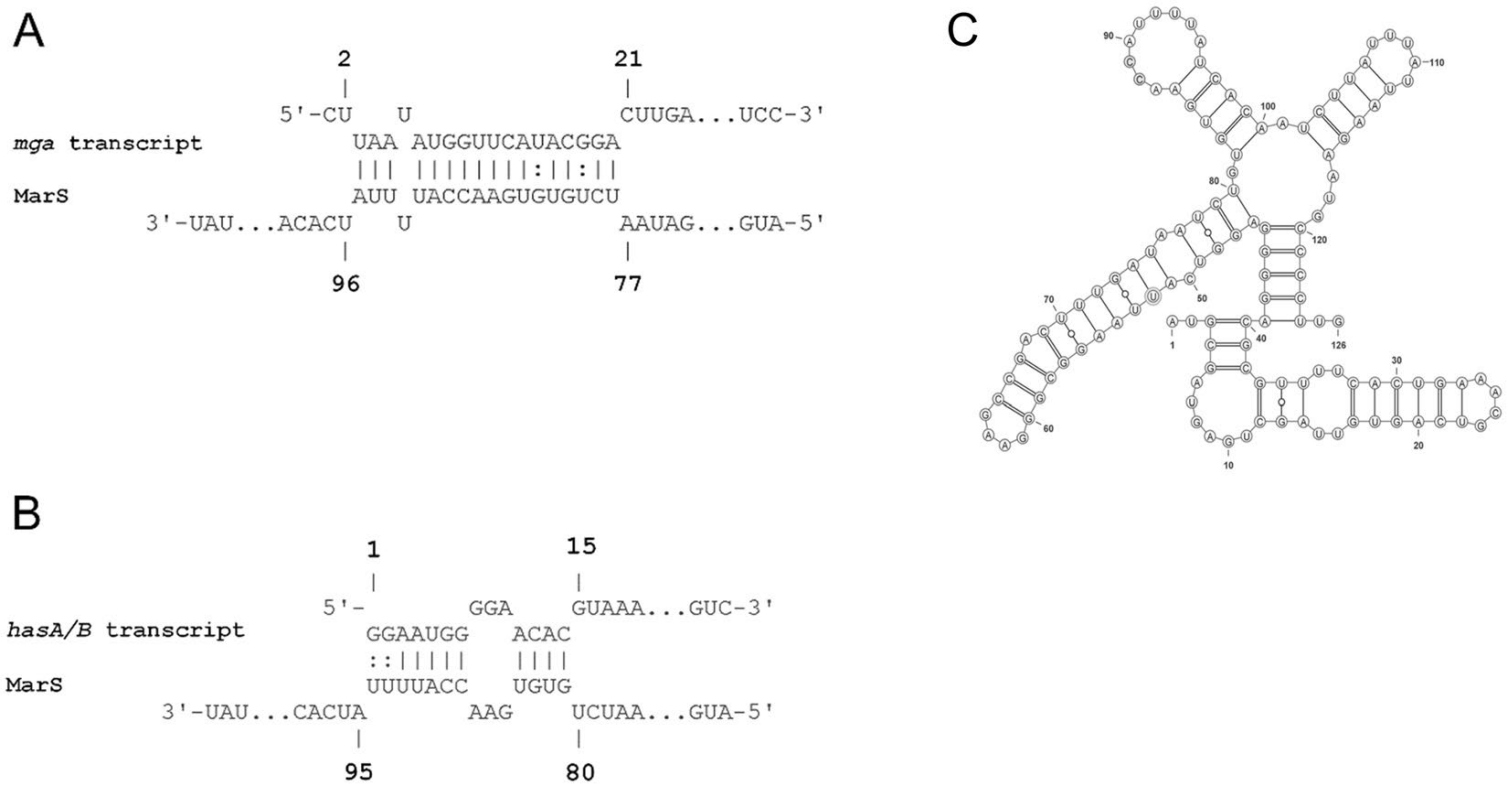
Putative interaction of MarS and its target transcripts. (**A**) Schematic of the putative binding of MarS to the *mga* transcript as predicted by IntaRNA. (**B**) Schematic of the putative binding of MarS to the *hasA/B*-transcript as predicted by IntaRNA. (**C**) Secondary structure predicted by RNAfold. Nucleotides 1–126 are depicted; the terminator stem loop is omitted for clarity.

One of the major mechanisms of sRNA-mediated regulation in bacteria is direct binding of the sRNA via base pairing to a target mRNA. Complex formation depends on complementary regions within the RNA sequences and on the respective secondary structures. The IntaRNA^30,31^ algorithm was employed to predict MarS-mRNA interactions in GAS M49 591. The sequence of *S. pyogenes* NZ131 (NC_011375.1) served as a reference genome. Among the 28 predicted targets, *mga*, the gene encoding the multiple virulence gene regulator, was identified with a high probability (Table 1). The putative binding site lies within the 5´untranslated region (5´UTR) of *mga* (Fig. 5A) and is conserved in serotypes M2, M4, M18, M28, M44, M49, M53, M59, M66, M71, M82, M83, M 87, M89, and M101 as determined by BLAST^23^ analysis. *In silico* prediction also identified *hasB*, encoding the UDP-glucose 6-dehydrogenase, which is part of the capsule synthesis operon in GAS (Table 1). The predicted binding site is located on the *hasABC* polycistronic transcript at the 3′ end of the *hasA* coding region (Fig. 5B). For both targets, a common binding site is situated in MarS. The secondary structure of MarS was predicted by RNAfold (The ViennaRNA Web Services, http://rna.tbi.univie.ac.at/) and illustrated using VARNA GUI^32^ (Fig. 5C).

**Table 1:** IntaRNA predictions.

Rank	p-value	fdr value	Target	Locus_tag	Gene	Energy
1	0.0003	0.46126	Spy49_1814	SPY49_RS08930		−12.06
2	0.0007	0.55764	Spy49_0125	SPY49_RS00815		−11.40
3	0.0013	0.70451	Spy49_1673c	SPY49_RS08270	mga	−10.89
4	0.0036	0.81466	Spy49_0999	SPY49_RS04990		−9.96
5	0.0040	0.81466	Spy49_0695c	SPY49_RS03545	mvaS2	−9.87
6	0.0043	0.81466	Spy49_0036	SPY49_RS00340		−9.80
7	0.0054	0.81466	Spy49_1678c	SPY49_RS08295		−9.59
8	0.0055	0.81466	Spy49_1719c	SPY49_RS08470	csp	−9.57
9	0.0063	0.81466	Spy49_1426c	SPY49_RS07050	rpsR	−9.45
10	0.0064	0.81466	Spy49_1639c	SPY49_RS08100	nudC	−9.42
11	0.0068	0.81466	Spy49_0502c	SPY49_RS02625		−9.37
12	0.0069	0.81466	Spy49_0898	SPY49_RS04510	glyA	−9.35
13	0.0071	0.81466	Spy49_1173c	SPY49_RS05800	ftsZ	−9.33
14	0.0072	0.81466	Spy49_1714c	SPY49_RS08445		−9.31
15	0.0075	0.81466	Spy49_0207	SPY49_RS01190	rnpA	−9.28
16	0.0077	0.81466	Spy49_0169	SPY49_RS01025		−9.24
17	0.0099	0.8874	Spy49_1320c	SPY49_RS06540		−8.99
18	0.0103	0.8874	Spy49_1674c	SPY49_RS08275		−8.96
19	0.0104	0.8874	Spy49_1443c	SPY49_RS07135		−8.94
20	0.0111	0.8874	Spy49_1578c	SPY49_RS07805	salY	−8.88
21	0.0113	0.8874	Spy49_1806	SPY49_RS08895	hasB	−8.86
22	0.0119	0.8874	Spy49_1655	SPY49_RS08175		−8.80
23	0.0121	0.8874	Spy49_0144	SPY49_RS00905	nga	−8.79
24	0.0128	0.90121	Spy49_1533c	SPY49_RS07550		−8.73
25	0.0142	0.90121	Spy49_1480c	SPY49_RS07300		−8.62
26	0.0152	0.90121	Spy49_1159c	SPY49_RS05730		−8.55
27	0.0171	0.90121	Spy49_0916c	SPY49_RS04600		−8.43
28	0.0193	0.90121	Spy49_1700c	SPY49_RS08390		−8.29

### MarS binds to the 5′ untranslated region of *mga*

**Figure 6:**
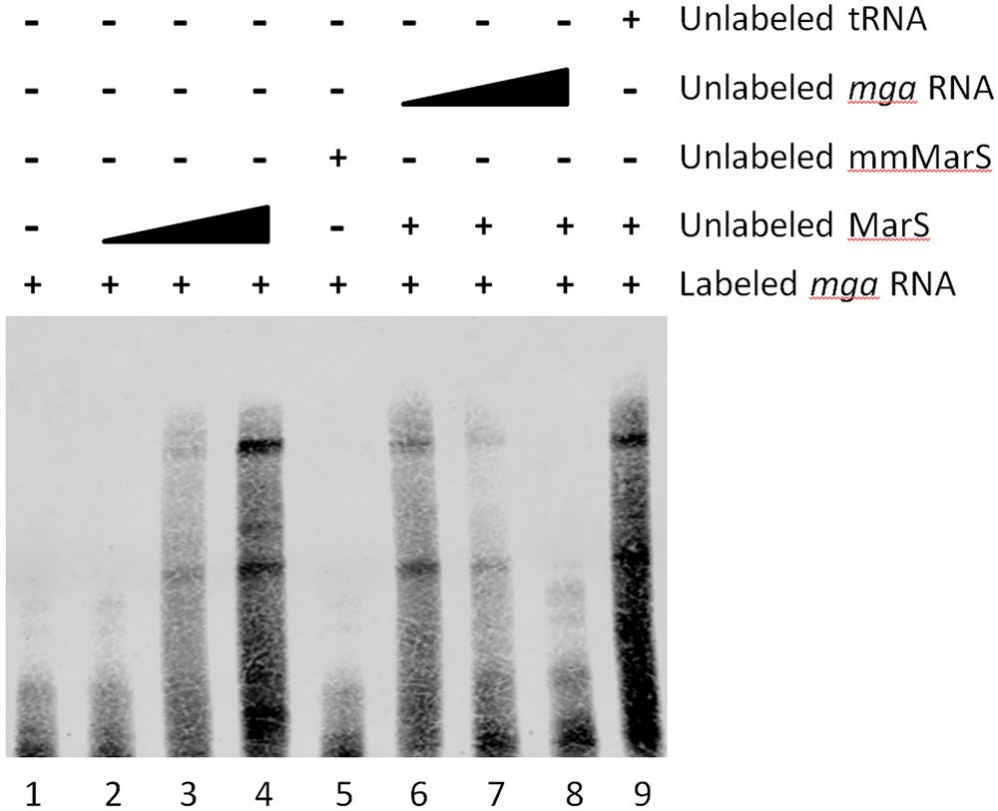
MarS binds to the 5′ UTR of *mga*. RNA-RNA EMSA verifying base pairing between MarS and the 5′UTR of *mga*. A biotin-labeled *mga* 5′ UTR RNA probe was incubated with wild-type MarS (lanes 2 to 4) or a MarS mutant (mmMarS), in which two bases situated in the putative binding site were exchanged (88-CC-89/88-GG-89) (lane 5). An unlabelled *mga* RNA probe was employed for cold competition (lanes 6–8), and unlabelled yeast tRNA was used as a specificity control (lane 9).

To determine whether MarS binds directly to the 5′ untranslated region of the mga mRNA, we performed RNA-RNA electrophoretic mobility shift assays (EMSAs). A biotin-labeled RNA corresponding to the 5′ end of the *mga* RNA was incubated with increasing amounts of MarS, which resulted in a shift of the probe to a higher molecular weight complex (Fig. 6, lanes 2-4). To verify the role of the predicted mRNA binding site in MarS (Fig. 5C), we used a mutated form of MarS (mmMarS), in which two residues of the putative binding site were replaced (88-CC-89/88-GG-89). In reactions containing mmMarS, the *mga* RNA probe was not shifted (Fig. 6, lane 5). The specificity of the binding was confirmed by incubation with increasing concentrations of unlabeled *mga* RNA, which reduced the shifting (Fig. 6, lanes 6-8).

### Deletion of *marS* results in lower *mga* and *mga*-controlled transcript levels

**Figure 7:**
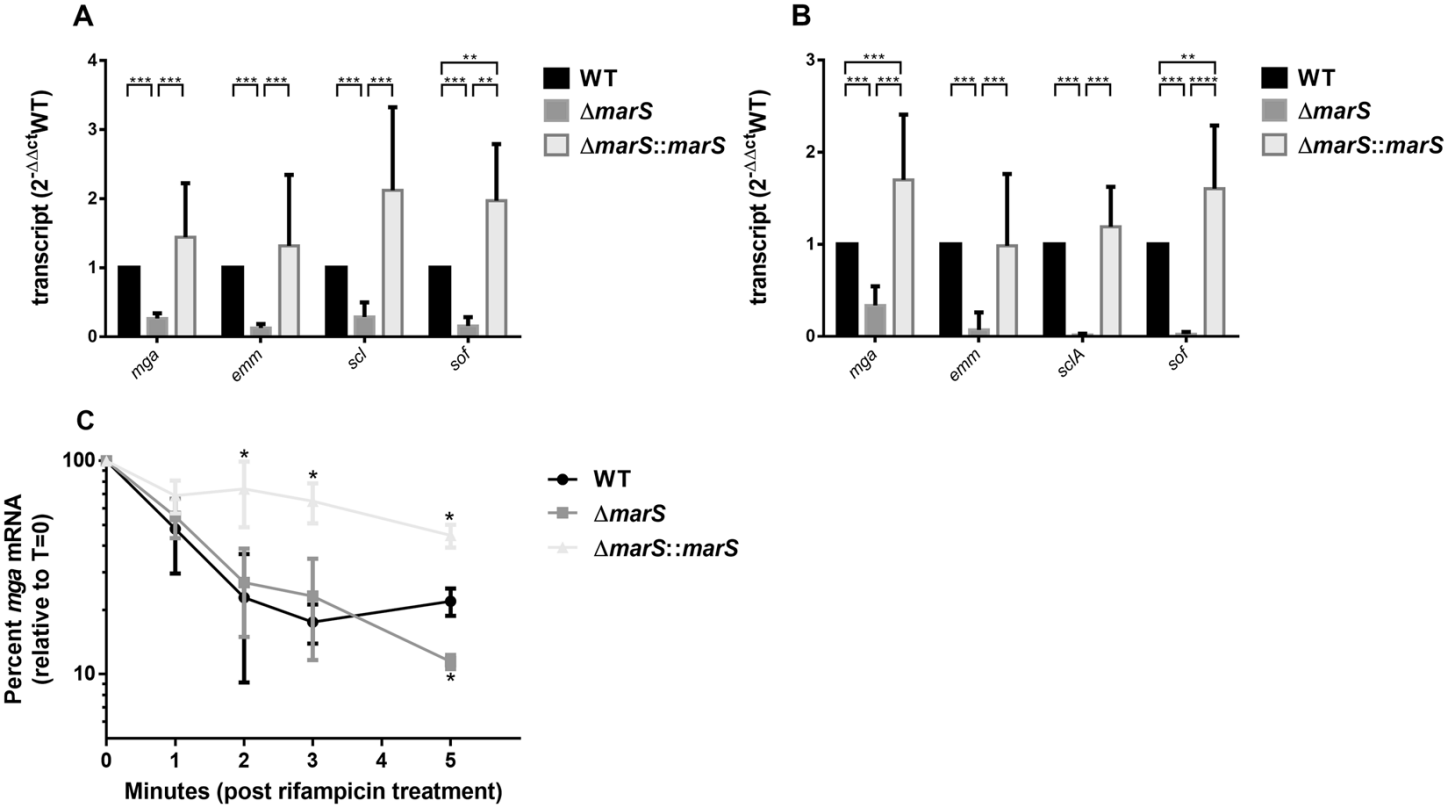
MarS influences *mga* and Mga-dependent transcript abundance. (**A**) Relative expression of *mga* and Mga-dependent transcripts in ∆*marS* (dark grey) and Δ*marS*::*marS* (light grey) in comparison to WT (black). Bacteria were grown in THY to the transitional growth phase, n = 8. (**B**) Relative expression of *mga* and Mga-dependent transcripts in ∆*marS* (dark grey) and Δ*marS*::*marS* (light grey in comparison to WT (black). Bacteria were exposed to human blood for 1 h, n = 8. The data are presented in comparison to WT as the mean values ± standard deviation. (**C**) Stability of *mga* transcript in ∆*marS* (dark grey) and Δ*marS*::*marS* (light grey) and WT (black) as determined by RT-qPCR following treatment of the culture with rifampicin; n = 3. Stability is presented as percent *mga* transcript level relative to time-point zero. The data are presented as the mean values ± standard deviation. The Student’s t-test was used to calculate statistical significance. Differences between samples are expressed as “not significant” (*P* ≥ 0.05) and marginally significant (*P* < 0.05)*.

The influence of MarS on the transcript abundance of the putative target *mga* and virulence factor genes directly regulated by Mga^33^ was investigated. Total RNA from GAS M49 591 and the recombinant derivatives either grown in THY (Fig. 7A) or following exposure to human blood (Fig. 7B) was analysed by RT-qPCR with primers specific for *mga*, *emm*, *sclA* (streptococcal collagen-like protein), and *sof* (serum opacity factor). In all cases tested, the abundance of the transcripts was significantly decreased in the deletion mutant compared to that in WT (Fig. 7A/B). In Δ*marS*:*:marS*, the phenotype could be restored. Together, these results indicate a positive regulatory influence of MarS on *mga* and thereby on the expression of genes regulated by Mga. To examine the impact of *mga* mRNA stability on this effect, the transcript abundance was determined following rifampicin treatment (Fig. 7C). The lack of MarS did not influence *mga* transcript stability within the first three min. In Δ*marS*:*:marS*, *mga* mRNA stability was slightly improved compared with that in WT and the deletion mutant. The half-life of the *mga* transcript as calculated by linear regression analyses was 0.57 min in WT, 1.15 min in *ΔmarS*, and 4.58 min in *ΔmarS::marS*. Thus, the effects on the Mga regulon in *ΔmarS* were not caused by reduced *mga* mRNA stability in the *marS* deletion strain.

### Down-regulation of MarS target gene transcription affects the proteome of GAS M49 591

Given the differential expression of several Mga-controlled genes encoding surface proteins in Δ*marS*, we set out to investigate the proteome of the sRNA deletion strain compared with WT and the complementation strain. Cytoplasm-depleted fractions were analysed by nano LC-mass spectrometry. The total number of proteins quantified by at least three and two peptides amounted to 885 and 1001, respectively, representing a high coverage of the proteome (Supplementary Data S1). Seventeen proteins were differentially regulated (a fold change above two) between the investigated strains (Table 2). For the majority of those, differential synthesis could be detected in the exponential (exp), transitional (tra), and stationary (stat) growth phases. Antiphagocytic M protein, fibronectin-binding protein, Fc-gamma receptor, and serum opacity factor, encoded by *emm49*, *sfbX49*, Spy49_1672c, and *sof*, respectively, were reduced in Δ*marS* samples compared with those in WT, whereas protein abundance was restored in the complementation strain (Table 2). Mga protein abundance was also reduced in the deletion strain in comparison to WT, but quantification was not meaningful in the cytosol-depleted samples that were used for the analyses. Mga contamination most likely resulted from an interaction with chromosomal DNA hampering complete removal from those samples. Thiol-activated cytolysin (*slo*) was reduced in Δ*marS* during exp and stat. In the complementation strain, the protein level was slightly increased in comparison to WT during tra and stat. Thus, down-regulation of the Mga-controlled gene transcription observed in Δ*marS* (Fig. 7A) resulted in a decreased synthesis of the corresponding gene products in the deletion mutant. Furthermore, the collagen-like surface proteins A and B and C5a peptidase were reduced in Δ*marS* samples in comparison to WT. The hyaluronic acid synthesis proteins HasA and HasB were increased in Δ*marS*:*:marS*, which is in accordance with the increased capsule production by this strain (Fig. 3A). Abundance of the cysteine proteinase SpeB was significantly reduced in both Δ*marS* and in Δ*marS*:*:marS* in comparison to WT, indicating that these changes were not MarS-dependent. Taken together, of 13 proteins that were down-regulated in Δ*marS* compared with those in WT, 10 were known surface-associated or secreted molecules, and two were proteins of unknown function. Although the extracellular ratios of the secreted proteins may differ from the values measured in the cytoplasm-depleted fraction of the cell extracts, their general regulation should be reflected by the data. The protein abundance of the capsule synthesis proteins HasA and HasB was significantly increased in Δ*marS*:*:marS* compared with those in WT.

**Table 2:** Differentially expressed proteins in the proteomes of GAS M49 591 WT, Δ*marS* and Δ*marS*::*marS*. Detailed data are provided in Supplementary Data S1.

Protein	Gene	Fold change (mean from three biological replicates)					
		WT/Δ*marS*			WT/Δ*marS*::*marS*		
		exp	tra	stat	exp	tra	stat
Protein abundance significantly reduced in Δ*marS*							
Immunogenic secreted protein	*isp2*	3.1	2.4	5.3	2.0	1.3	1.7
Collagen-like surface protein A	*sclA*	n.d.^a^	6.6	16.2	n.d.	0.7	0.8
C5a peptidase	*scpA*	21.2^b^	4.2	9.2	1.4^b^	1.0	1.0
Antiphagocytic M protein	*emm49*	10.7	11.1	7.5^c^	1.6	0.9	0.9
Fc-gamma receptor	Spy49_1672c	10.4	9.7	4.3	1.1	1.3	1.2
M protein trans-acting positive regulator	*mga*	117.2	62.5	45.2^c^	2.2	1.2	1.1
Fibronectin-binding protein	*sfbX49*	28.4	11.2	21.3	2.0	0.9	3.1
Serum opacity factor	*sof*	6.5^b^	3.7	9.5	3.1	1.1	1.6
Putative secreted protein	Spy49_0015	1.6^b^	2.6	1.3	1.5	2.0	0.8
Thiol-activated cytolysin	*slo*	3.1	1.1	3.2	1.1	0.4	0.6
Uncharacterized protein	Spy49_0343	n.d.	17.8^b^	8.6^b^	n.d.	1.0^b^	0.6^b^
Uncharacterized protein	Spy49_0412	n.d.	n.d.	8.7	n.d.	n.d.	0.8
Collagen-like surface protein B	*sclB*	4.7	5.4	5.3	1.1	1.0	0.9
Protein abundance significantly reduced in Δ*marS* and Δ*marS*:*:marS*							
Spi SpeB protease inhibitor	Spy49_1689c	n.d.	n.d.	2.3	n.d.	n.d.	6.9
Cysteine proteinase SpeB	*speB*	n.d.	2.4	5.2	n.d.	13.8	4.7
Protein abundance significantly increased in Δ*marS*:*:marS*							
Hyaluronan synthase	*hasA*	1.1	0.7	0.4	0.2	0.1	0.1
Putative UDP-glucose 6-dehydrogenase	*hasB*	1.2	1.5	1.2^b^	0.1	0.3	0.5^b^

^a^n.d.: missing values for quantification due to growth phase-dependent low protein amounts. ^b^Mean from two biological replicates. ^c^Mean was calculated from two biological replicates due to an infinite fold change in the third biological experiment (protein was not detected in Δ*marS*).

### Lack of MarS leads to a greater bacterial dissemination *in vivo*

**Figure 8:**
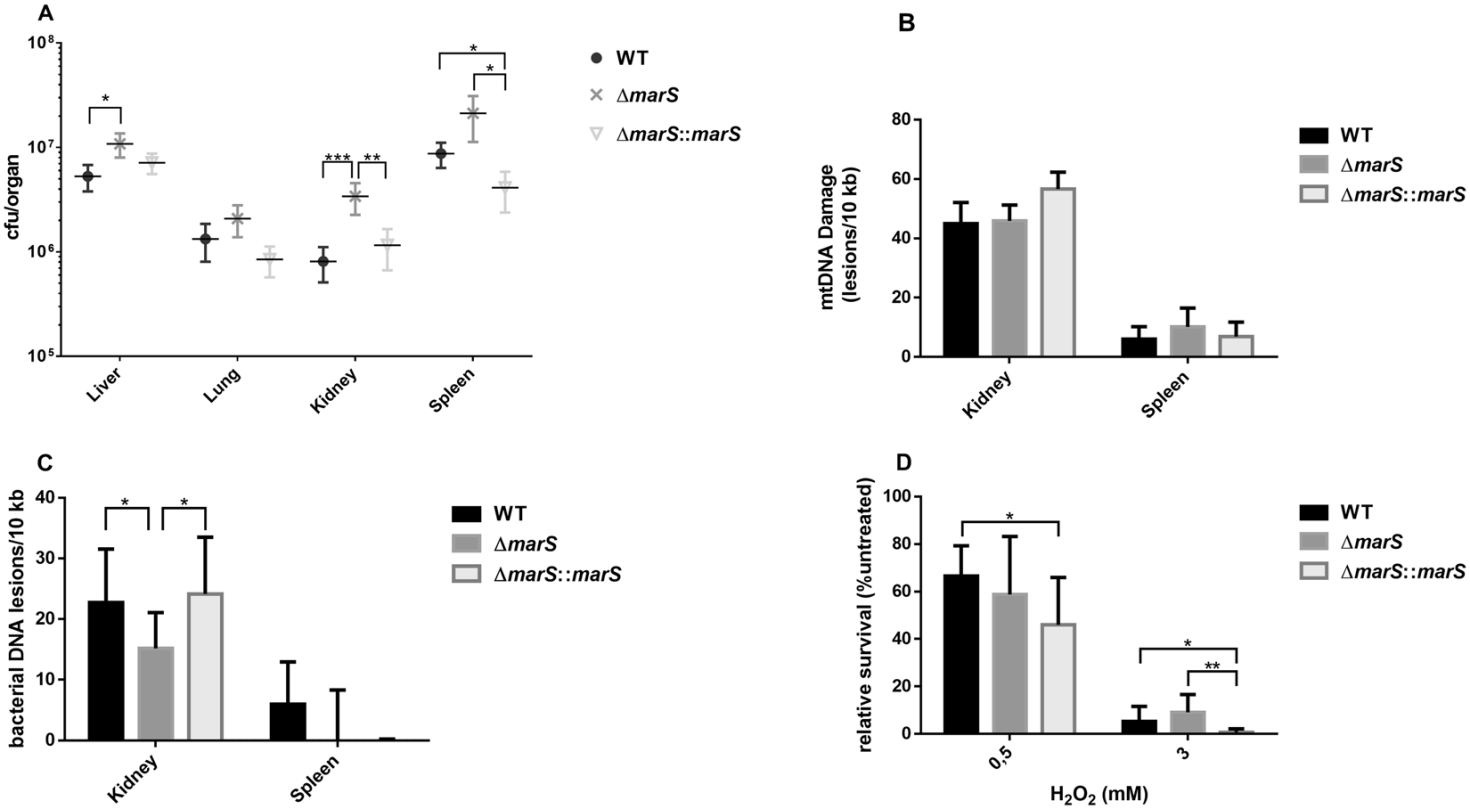
Deletion of *marS* leads to greater dissemination of GAS M49 591 in mice. (**A**) CFUs of ∆*marS* (dark grey) and Δ*marS*::*marS* (light grey) in comparison to WT (black) isolated from different organs 24 h after infection of BALB/c mice, n = 15 mice/group. (**B**) mtDNA damage of kidney and spleen tissue from infected mice, n = 3. (**C**) Bacterial DNA damage detected in kidney and spleen samples from infected mice, n = 6. (**D**) Survival of ∆*marS* (dark grey) and Δ*marS*::*marS* (light grey) and WT (black) following treatment with H_2_O_2_ as indicated; n = 8. The data are presented as the mean values ± standard deviation. Statistical significance for was determined using the two-tailed Mann-Whitney U test. Differences between samples were expressed as “ns = not significant” (P ≥ 0.05), marginally significant (P < 0.05)*, significant (P < 0.01)**, and highly significant (P < 0.0001)***.

To assess the influence of MarS on the virulence of GAS M49 591 *in vivo*, a mouse infection model was employed. Mice were infected i.p. with 8 × 10^7^ colony forming units (CFUs) GAS M49 591 WT, Δ*marS*, and Δ*marS*:*:marS*. All animals showed signs of severe bacterial infection and were sacrificed after 24 h. Bacteria were sampled from mouse liver, lung, kidney, and spleen for CFU determination. From the kidneys of infected mice, a significantly greater number of living Δ*marS* bacteria could be isolated compared with that in WT and the complementation strain (Fig. 8A). In the liver, significantly more Δ*marS* cells were detected than WT cells; in the spleen, the number of Δ*marS*:*:marS* bacteria was significantly reduced in comparison to the parental and deletion strains (Fig. 8A). A slightly larger number of Δ*marS* was detected in the lungs of the infected mice compared with that of the other two strains, but the differences between the samples were not significant. Thus, in all organs tested, there was a greater number of viable bacteria in the deletion strain than in WT and the complementation strain.

### Increased survival of Δ*marS* in the host kidney correlates to increased oxidative stress resistance

Reactive oxygen species (ROS)-related DNA damage was measured using a qPCR-based method^34^. Therefore, total DNA from the kidney and spleen of infected mice, containing host DNA and bacterial DNA, and of PBS-treated controls, containing only host DNA, was isolated. DNA lesions were detected in the mitochondrial DNA (mtDNA) of the host and in the genomic DNA of bacteria using primers specific for the mitochondrial D-loop sequence and the bacterial *gyrA*, respectively. Infection with any of the three strains resulted in a high level of mtDNA damage in the kidney (≥30 lesions per 10 kb) (Fig. 8B). The differences between the mtDNA damage of the samples collected following infection with the different strains were not significant. In the spleen, which showed a higher bacterial load in this experiment (Fig. 8A), mtDNA damage was less severe than in the kidney (Fig. 8B). Again, no strain-specific differences in mtDNA damage could be detected. Corresponding to the mtDNA data, bacterial DNA isolated from the kidney showed a high level of damage. Bacterial DNA damage was significantly reduced in Δ*marS* in comparison to the parental and the complementation strains (Fig. 8C). In contrast, the overall bacterial DNA damage in samples isolated from the spleen was low. DNA from WT cells showed approximately 5 lesions/10 kb, but the difference in comparison to untreated bacteria was not significant (Fig. 8C). Survival of the different strains under conditions of oxidative stress was tested *in vitro* using H_2_O_2_ as a stressor. Under high oxidative stress conditions (3 mM H_2_O_2_), the *marS* deletion strain showed significantly increased survival compared with that of the complementation strain (Fig. 8D). These data correspond to the situation in the kidneys of infected mice (Fig. 8A,B and C). Taken together, high oxidative stress in the host kidney correlated with a lower bacterial load. Under these conditions, bacterial DNA damage was reduced in Δ*marS* compared with that in WT and survival of the deletion mutant was facilitated.

## Discussion

In this study, we provide data regarding the role of the sRNA MarS in GAS M49 591 in the expression of virulence genes. GAS adheres to human tissue during colonization. Additionally, internalization by host cells has been discussed as a cause of recurrent disease, bacterial persistence in the host, and therapeutic failure of penicillin treatment^35–38^. Deletion of *marS* in GAS M49 591 resulted in a reduced adherence to human keratinocytes and a greater susceptibility to phagocytosis. The M protein supports evasion of phagocytosis^25,26,39,40^ and is involved in adherence and internalization processes^37,41,42^. Expression of *emm*, which encodes the antiphagocytic M protein, was dramatically reduced in ∆*marS*, explaining the phagocytosis and the adherence phenotype of the deletion strain. Activation of *emm* expression is controlled by Mga. The *mga* mRNA, which was one of the MarS targets predicted by IntaRNA was also decreased in the deletion strain. In the case of a direct interaction of MarS with the predicted binding site located at the 5′-end of the *mga* transcript, translation may be diminished in the deletion strain. In turn, decreased protein levels of Mga would lead to hampered stimulation of *mga* transcription by Mga. The expression of *mga* is positively autoregulated^43^. A small amount of *mga* transcript in ∆*marS* is consistent with the down-regulation of several genes that are known to belong to the Mga regulon^33^, including *sclA* and *sof*. A low abundance of the collagen-like surface proteins A and B, and C5a peptidase was observed in the proteome of ∆*marS*. Expression of the corresponding genes *sclA*, *sclB*, and *scpA* is known to be transcriptionally controlled by Mga^44–46^. We conclude that an influence of MarS on *mga* expression leads to down-regulation of *mga* in the deletion strain and, consequently, a down-regulation of Mga-activated genes. Direct binding of MarS to the predicted *mga* mRNA binding site was supported by EMSAs. The *marS* gene is conserved throughout GAS, and the binding site in the 5′ UTR of *mga* is conserved in several GAS *emm* types as determined by BLAST^23^ analysis. Amongst others, these include *emm18*, *emm28*, *emm*59, and *emm83*, which are responsible for diseases as diverse as acute rheumatic fever, puerperal sepsis, severe invasive disease, and skin tissue infections^47–50^. Binding of sRNAs to their target mRNAs is a typical characteristic of trans-acting sRNAs in many bacteria. Recently, direct sRNA-mRNA binding has also been observed in GAS. The interaction of FasX with its targets mRNAs *prtF1* and *prtF2* and *cpa* could be observed *in vitro* by gel shift assays^17,18^. FasX mediated regulation of pilus genes occurred in a serotype-specific manner^16^. Accordingly, target prediction in different GAS genomes in combination with *in vitro* binding studies might lead to the discovery of an *emm*-type specific target spectrum of MarS.

Depending on the GAS serotype, the infection model used, and the nature of the mutation, virulence of surface protein-deficient GAS strains in mice is affected differently. In a recent study, a commonly occurring single-nucleotide polymorphism (SNP) in GAS M59 increased expression of *mga* and 54 other genes, leading to significantly larger skin lesions in mice^51^. Decreased Mga activity or inactivation of *mga* or *emm*, respectively, led to attenuated virulence of GAS in mouse models for skin infection^52–54^. While fibronectin binding promoted bacterial adherence, dissemination to the spleen of infected mice was less efficient in GAS expressing fibronectin-binding protein F1 in comparison to bacteria lacking this protein^55^. Disruption of pilus assembly by sortase deletion rendered the GAS serotype M49 significantly more aggressive in a dermonecrotic mouse infection model^56^. Taken together, the down-regulation of surface-bound virulence factors leads to hampered adherence. Under these circumstances, in systemic infection models, bacterial dissemination is promoted. In our model, deletion of *marS* led to down-regulation of several surface proteins, including the M protein and the fibronectin-binding proteins SfbX49 and Sof. Consequently, the ability to adhere to keratinocytes was diminished, while dissemination in a sepsis mouse model was increased.

One prerequisite for successful infection is the ability of the pathogen to withstand the oxidative stress conditions generated by the host at the site of infection. GAS employs a variety of resistance mechanisms towards ROS, including physical barriers, enzymatic reactions, and metal homeostasis^57^. Bacterial DNA damage in ∆*marS* was decreased compared with that in WT under high oxidative stress conditions in the kidney, indicating that MarS influences the expression of genes involved in the oxidative stress response; however, the regulatory target remains unknown. In this context, a lack of MarS was advantageous for GAS M49 591-survival in the host.

In the proteomes of Δ*marS* and in Δ*marS*:*:marS*, the abundance of the cysteine proteinase SpeB was significantly reduced compared with that in WT. SpeB production is regulated by the CovR/S two-component system and plays a specific role in invasive disease. Comparative genomics and transcriptomics revealed that invasive GAS M1 strains exhibit a SpeB switch caused by CovR/S mutations, leading to the increased expression of several virulence factor genes, including *ska, slo, sda1, sic, and scpA*, and to decreased SpeB abundance^58,59^. SpeB production is also stimulated by Mga in GAS M49 591, but there is no direct binding of the *speB* promoter by Mga^46^. The down-regulation of SpeB in ∆*marS* could be caused by decreased levels of Mga in this strain. However, the phenotype was not complemented by ectopic *marS* expression from a plasmid, indicating a more complex situation.

One of the putative targets identified by the IntaRNA algorithm was *hasB*, encoding the UDP-glucose 6-dehydrogenase, which is part of the capsule synthesis operon in GAS. There were no significant differences in hyaluronic acid content between WT and Δ*marS*, whereas Δ*marS*:*:marS* produced significantly more capsule in comparison to the WT strain. Although the hyaluronic acid capsule is known to impact resilience to neutrophils^25,26^, differential capsule production was not observed in the deletion mutant and did not lead to decreased survival in the phagocytosis assay. The capsule is also known to influence the adherence of GAS to human keratinocytes^42,60^. In our model, ∆*marS* could not bind as efficiently as WT to HaCaT cells. Δ*marS*:*:marS* was able to restore the WT phenotype, but the high hyaluronic acid content of the strain did not lead to significantly increased adherence. Therefore, hyaluronic acid does not seem to play a major role as an adhesin in this serotype. GAS M49 591 naturally produces small amounts of hyaluronic acid in comparison to other serotypes. Consequently, we observed a more pronounced regulatory effect of MarS on capsule production in GAS M18 MGAS8232.

Together, MarS modulates the expression of virulence factor genes belonging to the Mga regulon and influences hyaluronic acid production in GAS, thereby promoting virulence. While FasX is a negative regulator of pili and the fibronectin-binding proteins PrtF1 and PrtF2, it upregulates the expression of streptokinase, thereby functioning as a switch from colonization to dissemination^17,18^. In contrast, MarS promotes adhesion by enhancing the expression of several extracellular matrix-binding surface proteins and stimulating capsule production while suppressing bacterial dissemination. We propose that MarS is involved in GAS colonization during the early stages of infection.

## Methods

### Bacterial strains and culture conditions

The GAS serotype M49 strain 591 was kindly provided by R. Lütticken (Aachen, Germany). The GAS serotype M18 strain MGAS8232 was obtained from the Centre of Epidemiology and Microbiology, National Institute of Public Health, Prague, Czech Republic. All GAS strains were cultured in chemically defined medium (CDM)^61^ or Todd-Hewitt broth (Thermo Fisher Scientific, Darmstadt, Germany) supplemented with 0.5% yeast extract (Thermo Fisher Scientific, Darmstadt, Germany) (THY), as indicated, at 37 °C with a 5% CO_2_/20% O_2_ atmosphere. *Escherichia coli* strain DH5α (Gibco BRL, Eggenstein, Germany) was used as a host for the construction, proliferation, and storage of recombinant plasmids. All *E. coli* strains were cultured in Lennox L Broth Base (Thermo Fisher Scientific, Darmstadt, Germany). For selection, antibiotics were added at the appropriate concentrations.

### Construction of recombinant GAS strains

For the construction of an isogenic *marS* deletion mutant of GAS M49 591 (Δ*marS*) and GAS M18 MGAS8232 (GAS M18 MGAS8232 Δ*marS*), *marS* was exchanged for a spectinomycin resistance cassette by homologous recombination^62^. To supply sequence to mediate homologous recombination, an upstream 800 bp flanking region 1 fragment and a downstream 931 bp flanking region 2 fragment were amplified by PCR using chromosomal DNA from the parental strain as a template. All primers used for the generation of the respective fragments are listed in Supplementary Table S1. PCR products were sequentially cloned into the MCS of pUC18Erm1^63^. Between the flanking regions, a spectinomycin resistance cassette from pFW5^62^ was cloned into the BamHI site. The resulting suicide plasmid was verified by classical Sanger sequencing (GATC Biotech AG, Konstanz, Germany) and was used to transform GAS strains M49 591 or M18 MGAS8232. Deletion of *marS* was confirmed by sequencing. Therefore, a PCR product of the genomic region was analysed by classical Sanger sequencing (GATC Biotech AG, Konstanz, Germany). Loss of MarS was determined by RT-qPCR. For construction of a complementation strain (Δ*marS*:*:marS*), a fragment including the endogenous promotor and terminator regions was amplified by PCR and cloned into the SalI/BamHI sites of the shuttle vector pAT19^64^. The resulting vector was verified by sequencing and used for transformation of Δ*marS*. The absence of spurious mutations in the recombinant strains was confirmed by MiSeq whole genome sequencing. The corresponding data were submitted to the European Nucleotide Archive (ENA/SRA) (Accession number: PRJEB18537).

### Assays to assess the ability of the bacteria to survive in blood and plasma

The ability of the bacteria to survive in blood (short: blood survival assay) was assessed as described by Nakata *et al*.^56^. Briefly, overnight cultures of the GAS parental strain and isogenic mutants were inoculated into fresh medium and grown to the exponential growth phase. The cultures were centrifuged, washed and suspended in phosphate-buffered saline (PBS). Next, 20 µl of each respective cell suspension was used to inoculate 480 µl heparinized human blood. Blood was obtained from at least three individual volunteers. The samples were incubated for 3 h at 37 °C under rotation. CFUs of the samples were determined following serial dilution and plating on THY agar plates and compared to the CFUs of the respective inoculate. The resulting multiplication factor (MF) was used to compare strains. The data were normalized by setting the MF of the WT to 100%. The ability of the bacteria to survive in plasma (short: plasma survival assay) was assessed accordingly using human plasma as the medium.

### Quantitative phagocytosis assay

Human neutrophils were isolated from human blood using PolymorphPrep™ (PROGEN Biotechnik GmbH, Heidelberg) according to the instructions of the manufacturer. The neutrophils were suspended in RPMI 1640 (Invitrogen, Thermo Fisher Scientific, Darmstadt, Germany). Bacteria were grown over night and washed with PBS. For opsonization 10^7^ CFU/ml were incubated for 20 min with 10% human serum at room temperature. The opsonized bacteria were incubated with 10^7^ human neutrophils/ml (1:1) and 5% serum for another 30 min at 37 °C. As a reference, a sample of the opsonized bacteria was incubated without neutrophils. To determine the survival rate, the samples were centrifuged and the pellets lysed in sterile distilled water. The counts of viable GAS were determined following serial dilution and plating on THY agar. To determine the proportion of extracellular bacteria, the samples were centrifuged for 5 min at 100 g for separation. The supernatant was collected, centrifuged at 13.000 g, and the bacterial pellet was dissolved in PBS.

### Quantification of hyaluronic acid

The amount of cell-associated hyaluronic acid produced by GAS was determined by the release of capsule from cells in the exponential growth phase and subsequent measurement of the hyaluronic acid content using Stains-All (Sigma) as described previously^37^. The absorbance at 640 nm was compared to a standard curve prepared with known concentrations of hyaluronic acid. The amount of hyaluronic acid in the samples was calculated in fg/CFU. The data are expressed relative to the content of hyaluronic acid in the respective WT strain.

### Adherence and internalization assay

Bacterial adherence to and internalization into the human keratinocyte cell line HaCaT (DKFZ, Heidelberg, Germany) was quantified employing an infection assay^65^. In brief, 24-well-plates were inoculated with 2.5 × 10^5^ HaCaT cells per well in DMEM (Invitrogen, Thermo Fisher Scientific, Darmstadt, Germany) without antibiotics. Growth was allowed until confluence. Keratinocytes were washed with DMEM and infected separately with GAS strains of interest in DMEM at a multiplicity of infection (MOI) of 1:10. After 2 h incubation at 37 °C in a 5% CO_2_ atmosphere, the keratinocytes were washed extensively with PBS. To detach the keratinocytes, trypsin/EDTA (Invitrogen, Thermo Fisher Scientific, Darmstadt, Germany) was added and the keratinocytes were lysed in sterile distilled water. CFU from GAS attached to and internalized into HaCaT keratinocytes were determined following serial dilution in PBS and plating on THY agar.

### Electrophoretic mobility shift assay (EMSA)

RNA:RNA EMSAs were performed as described by Danger *et al*.^18^. Briefly, RNA for RNA:RNA gel shift assays was prepared by *in vitro* transcription of the T7 promoter sequence containing PCR products using T7 polymerase. All primers used for the generation of the respective fragments are listed in Supplementary Table S1. Chromosomal DNA of GAS M49 591 served as the template for the *mga* 5′ region and the *marS* fragments and pAT19_ *marS* 88-CC-89/88-GG-89 served as the template for the mismatch *marS* fragment. *In vitro* transcription reactions were performed using the MEGAshortscript kit (Thermo Fisher Scientific, Darmstadt, Germany) according to the instructions of the manufacturer. Template DNA was removed using TURBO DNase (Thermo Fisher Scientific, Darmstadt, Germany). The RNA was purified using the RNA Clean and Concentrator-25 kit (Zymo Research Europe, Freiburg, Germany). For probe labelling, *mga* mRNA was biotin-labelled using the Pierce RNA 3′ end biotinylation kit (Thermo Fisher Scientific, Darmstadt, Germany). Probes were purified using the RNA Clean & Concentrator-5 kit (Zymo Research Europe, Freiburg, Germany). All RNAs were quantified using the Qubit 3.0 Fluorometer (Thermo Fisher Scientific, Darmstadt, Germany) and their quality was evaluated using the Agilent Bioanalyzer 2100 system. Labelled *mga* RNA (12 nM) was incubated in the presence or absence of MarS RNA (0, 10, 100, 1000 nM), mmMarS (1000 nM), unlabelled *mga* RNA (10, 100, 1000 nM) or unlabeled yeast tRNA (1000 nM). EMSA reactions (10 µl) contained 1 µl tRNA (10 µg/µl stock) and 1 µl of structure buffer (10 x stock; provided with the Lightshift RNA EMSA kit, Thermo Fisher Scientific, Darmstadt, Germany). Reactions were heated to 56 °C for 5 min before cooling to 37 °C for 30 min to allow refolding. REMSA loading buffer (Lightshift RNA EMSA kit, Thermo Fisher Scientific, Darmstadt, Germany) was added to each sample. The samples were separated on a 5% TBE mini-gel in 0.5 × TBE buffer, transferred by semi-dry blotting to a positively charged nylon membrane, and UV-crosslinked. The membrane was then blocked for one hour at RT (Odyssey Blocking Buffer, Li-Cor, diluted 1:3 in PBS), incubated with Streptavidin IRDye (Li-COR) at room temperature for 20 min in the dark, washed 3 x (PBST, 1% SDS), and the labeled RNA was detected using a Li-Cor Odyssey system. The membranes were rinsed and stored in PBS.

### Transcript stability determination

GAS strains were grown to the transitional growth phase in THY (OD_600_ of 0.8) before the addition of rifampicin (to 1 mg/ ml) to inhibit RNA synthesis. Following the addition of rifampicin, 10 ml samples were recovered after 0, 1, 2, 3, 5, 10, and 15 min, as indicated. Samples were pelleted by centrifugation and quickly frozen in liquid nitrogen. Total RNA was extracted as described below.

### Reverse transcription followed by quantitative PCR (RT-qPCR)

GAS strains were grown to the transitional growth phase in THY (OD_600_ of 0.8). Bacterial cells were either pelleted immediately and quickly frozen in liquid nitrogen or exposed to human blood for one hour prior to sample preparation. Total RNA from GAS strains was extracted according to the protocol supplied with the Direct-zol^TM^RNA MiniPrep Kit (Zymo Research, Irvine). After extraction, RNA was treated with acid phenol:chloroform:isoamyl alcohol (125:24:1), pH 4.5 (Thermo Fisher Scientific, Darmstadt, Germany), and TURBO^TM^DNAse (Thermo Fisher Scientific, Darmstadt, Germany) according to the manufacturer’s instructions. cDNA synthesis was performed using the Superscript first-strand synthesis system for RT-PCR (Invitrogen, Thermo Fisher Scientific, Darmstadt, Germany). Quantitative PCR amplification was performed with SYBR green (Thermo Fisher Scientific, Darmstadt, Germany) using the ViiA™ 7 Real-Time PCR System (Applied Biosystems, Darmstadt, Germany). The 5 S rRNA gene and the DNA gyrase subunit A gene (*gyrA*) served as housekeeping genes. Relative expression was calculated employing the 2^−ΔΔct^ method^66^. All primers used for RT-qPCR are listed in Supplementary Table S1.

### Northern blot analyses

Total RNA was isolated from GAS strains grown to the transitional growth phase in THY (OD_600_ of 0.8) as described above. RNA samples (10 μg) were loaded onto an 8% TBE-Urea polyacrylamide gel and separated by electrophoresis. Size standards (Ultra Low Range Ladder, Fermentas) were loaded on the same gel. RNA was electroblotted onto positively charged nylon membranes (Ambion) and UV cross-linked. Templates for the probes were generated by PCR with the same primers that were used for the PCR reaction in the RT-qPCR experiments. To the 5′ end of the reverse primers was added the T7 promoter sequence (CTTAATACGACTCACTATAGGG) for *in vitro* transcription (MAXIscript™ T7 Transcription Kit, Ambion). Probes were labelled with biotin prior to hybridization (Brightstar Psoralen-Biotin Labeling kit, Ambion). Membranes were hybridized overnight with a RNA probe complementary to MarS or 5 S RNA, as indicated. A BrightStar BioDetect Kit (Ambion) was used for detection, and autoradiography films were exposed to the luminescent blots.

### Extract preparation for proteome analyses

Bacteria were grown in THY and samples were collected at different time points during growth. Crude extracts were prepared in a precellys 24 homogenizer (peqLab Biotechnologie GmbH, Erlangen, Germany) and divided into cytoplasm-depleted and cytoplasmic fractions by centrifugation at 13000 g. Three biological replicates were performed.

### Proteome analyses

Mass spectrometry was performed on a Synapt G2-S mass spectrometer coupled to a nanoAcquity UPLC system (Waters, Manchester, UK). Peptides of the tryptic digests were separated by reversed-phase UPLC and analysed in data-independent mode (HDMS^E^). Label-free protein quantification and expression analysis were performed using Progenesis QI for Proteomics (Nonlinear Dynamics, Newcastle upon Tyne, UK). A detailed description of the experimental procedures is provided as supporting information (Supplementary Methods S1).

### Murine infection model

Naïve, inbred, 8-week-old female BALB/c mice were purchased from Charles River Laboratories (Sulzfeld, Germany). Mice were inoculated intraperitoneally with 8 × 10^7^ CFU of GAS strains, as indicated, in 0.2 ml PBS utilizing a BD Microlance 27 G 3/4” (Becton Dickinson GmbH, Heidelberg, Germany). Mock infection was performed with 0.2 ml PBS. As a vector control mice were infected with GAS M49 591 carrying pAT18/GFP. Twenty-four h post-infection, the mice were sacrificed using a ketamine/xylazine combination. Bacterial dissemination was investigated by determination of bacterial loads in different organs^67^. All the experimental protocols were approved by a licensing committee as specified in the ethics statement.

### Bacterial oxidative stress resistance

GAS strains were grown in THY to the early exponential growth phase. H_2_O_2_ (Roth, Karlsruhe, Germany) was added as indicated. Following incubation for 2 h at 37 °C, cells were transferred to ice, harvested, washed twice with PBS and plated on THY agar for CFU determination.

### DNA damage detection

Total DNA from mouse tissues was isolated using the DNeasy Blood and Tissue Kit (Qiagen, Hilden, Germany) according to the manufacturer’s instructions. DNA damage detection was performed as described elsewhere^34^. In brief, DNA quantity and purity were determined by spectrophotometric analysis. DNA lesion rates were determined by sequence-specific qPCR of mouse mitochondrial DNA (mmtDNA) and bacterial DNA, respectively. Amplification of a long (mmtDNA: 618 bp, *gyrA*: 775 bp) and a short (mmtDNA: 87 bp, *gyrA*: 85 bp) fragment was performed relative to DNA isolated from untreated cells. The small amplicon served as an undamaged DNA reference and allowed for DNA concentration normalization. PCR was performed with SYBR green (Thermo Fisher Scientific, Darmstadt, Germany) using a Light Cycler® 480 Instrument (Roche Diagnostics, Mannheim, Germany). All primers used for qPCR are listed in Supplementary Table S1. DNA was isolated from at least four animals, and qPCR reactions were performed in triplicates.

### Statistical Analyses

All experiments were performed at least three times or as indicated by the sample size (n). Statistical significance was determined for normalized data using the Wilcoxon signed-rank test. RNA stability tests were performed in three biological replicates; thus, the Student’s t-test was used to calculate statistical significance. For all other experiments, the test used to determine statistical significance is indicated in the respective figure legend.

### Ethics statement

The protocol for the collection of human blood for the blood survival assay was approved by the *Ethikkommission an der Medizinischen Fakultät der Universität Rostock* (ethics committee vote: A 2014-0131). The experiments were conducted in accordance with the ICH-GCP guidelines. Informed consent was obtained from all subjects. The protocol for the murine infection model was approved by the *Landesamt für Landwirtschaft, Lebensmittelsicherheit und Fischerei M-V* (Permit Number: 7221.3-1.1-090/12). Mice were sacrificed using a ketamine/xylazine combination. Animal experiments were performed in strict accordance with the German regulations of the Society for Laboratory Animal Science *(GV-SOLAS)* and the European Health Law of the Federation of Laboratory Animal Science Associations (FELASA).

### Data availability

The whole genome sequencing datasets generated during the current study are available in the European Nucleotide Archive (ENA/SRA) repository (Accession number: PRJEB18537). The proteome data generated during this study are included in this published article (and its Supplementary Information files). All additional datasets generated during or analysed during the current study are available from the corresponding author upon reasonable request.

## Electronic supplementary material

Supporting information

Dataset 1
