# Supplementary material for: The Regulatory Small RNA MarS Supports Virulence of Streptococcus pyogenes
Source: Sci Rep. 2017 Sep 25;7:12241. doi: 10.1038/s41598-017-12507-z (PMC5613026; doi:10.1038/s41598-017-12507-z)
Supplement: Supplementary file 1 — Supporting information [file 41598_2017_12507_MOESM1_ESM.pdf]

The Regulatory Small RNA MarS Supports Virulence of *Streptococcus pyogenes*

Roberto Pappesch[1], Philipp Warnke[1], Stefan Mikkat[2], Jana Normann[1], Aleksandra Wisniewska-Kucper[1], Franziska Huschka[1,3], Maja Wittmann[1], Afsaneh Khani[1], Oliver Schwengers[4,5], Sonja Oehmcke-Hecht[1], Torsten Hain[4], Bernd Kreikemeyer[1], Nadja Patenge[1*]

[1]Institute of Medical Microbiology, Virology and Hygiene, University Medicine Rostock, Rostock, Germany

[2]Core Facility Proteome Analysis, University Medicine Rostock, Rostock, Germany

[3]Present address: Franziska Huschka, Institute for Medical Microbiology, Virology and Hygiene, University Medical Center Hamburg-Eppendorf, Hamburg, Germany.

[4]Institute for Medical Microbiology, Justus-Liebig University of Giessen, Giessen, Germany

[5]Institute for Medical Microbiology, Justus-Liebig University of Giessen, Giessen, Germany

[*]Address correspondence to Nadja Patenge, nadja.patenge@med.uni-rostock.de

Supporting information

Supplementary Table S1. Sequences of oligonucleotides used for PCR.

Supplementary Methods S1. Experimental procedures for proteome analyses.

Supplementary Figure S1. Non-normalized data of bacterial growth in human blood.

Supplementary Figure S2. Non-normalized data of bacterial growth in human plasma and of bacterial survival in the presence of neutrophils.

Supplementary Figure S3. Original Northern blot X-ray film scan.

Separate supporting Data file:

Supplementary Data S1. Results of label-free quantification of protein abundances.

Table S1: Sequences of primers used for PCR

Primer name	Forward strand (5'->3')	Reverse strand (5'->3')
Sequences of primers used for vector construction		
flanking region 1		
Cand26-1	TACGTCGACGGGAGGTATCATTCACTGGA	TGGATCCATCGACTCATCGCATACAGAT
flanking region 2		
Cand26-2	GAGGATCCATGCCCCTTGGATTGAGATA	CGAGCTCCCAATCTTCAATGCCTGGAT
spectinomycin resistance cassette		
Spec-3	TGGGATCCGAAGCCAATGAAATCTATAAATAA	GAGGATCCGCGCTTACCAATTAGAATGAA
complementation strain fragment		
compl26	TGCGTCGACTTAGTACGAACGATTGCCC	AGGATCCGATACATTCTGAATTTTAACTC
Sequences of primers used for RT-qPCR		
5 S RNA	AGCGACTACCTTATCTCACAG	GAGATACACCTGTACCCATG
sRNASpy490957c (*marS*)	GATTATCAAAGTCGGCTTCCCGC	AGTCACTTTTGCGCAGGGGAGG
gyrA	CGACTTGTCTGAACGCCAAA	TTATCACGTTCCAAACCAGTCAA
mga	TTCACAGATAACAACGTTATGGTC	TTCTGGTTTTTGTACCTCTTTGTCACT
hasB	CAGAATCGAGAAAATTAAATAGTCACATG	AGACAATAACCTCCATAACCAAATGA
emm49	CGGAGAATAACGTGTCTAGC	CTGTAAGATCGGCGATTTGG
sclA	AAAACCTGCTGACAAAGAAGCTAAC	TGGCTACAGGTGTCTTAGCCATATT
sof	CAGTTCGATTATACCAAGCGTGTAG	TGAGGGCTACTACTACCACAGTTATT
Sequences of primers used for DNA damage determination		
gyrA long fragment	GGGATGGCAACTAACATTCCG	TCCAAACCAGTCAAACGACGC
gyrA short fragment	GCTCAGACAGAATTGATGTCA	TCCAAACCAGTCAAACGACGC
mmtD-loop long fragment	CCATATGACTATCCCCTTCC	GATTAGAGTTTTGGTTCACGG
mmtD-loop short fragment	AAGGACAGCACACAGTCTAG	CTTAGGTGATTGGGTTTTGCG
Sequences of primers used for EMSA probes		
T7_mga5prime	CTTAATACGACTCACTATAGGGTTTCCTTAATATGGTTCATACGGAC	CATGCATTAACTTCATGTCC
T7_marS	CTTAATACGACTCACTATAGGGATGCGATGAGTCGATTGTGACTGC	AAAAAAGGATTGAGAATATCTCAATCC

Experimental procedures for Proteome analyses

In-solution digestion of proteins

The cytoplasm-depleted sample pellets were mixed with 200 µl of solubilization buffer (50 mM ammonium bicarbonate (ABC), 1.5% sodium deoxycholate (SDC), 10 mM dithiothreitol (DTT)), incubated at 95 °C for 5 min, and subsequently sonicated for 10 min using a bath sonicator. Following centrifugation (22130 x g, 10 min at room temperature) 130 µl of supernatant was transferred into a new tube and alkylated with 15 mM iodoacetamide (IAA) for 20 min at room temperature. After a further centrifugation step, the supernatant was diluted with two volumes of 50 mM ABC. Sequencing grade trypsin (Promega GmbH, Mannheim, Germany) was added in an enzyme/protein ratio of approximately 1:100 to a final volume of 320 µl. Digestion was performed at 37 °C for 16 h.

For the digestion of the cytoplasmic fraction, 100 µg of protein in a final volume of 300 µl buffer containing 50 mM ABC, 0.5% SDC and 10 mM DTT were incubated for 5 min at 95 °C. Alkylation of proteins and digestion with trypsin was performed as described above. SDC was removed from the solutions of digested cytoplasmic and cytoplasm-depleted samples, respectively, using the phase transfer surfactant method (1). 320 µl of ethylacetate (1 vol) and 6.5 µl of 25% trifluoroacetic acid (0.5% final concentration) were added to the digested solution. The mixture was rigorously shaken for 2 min and subsequently centrifuged at 12000 x g for 10 min to obtain aqueous and organic phases. 200 µl of the aqueous phase was collected using a gel loading tip. Aliquots of the peptide solution were desalted with C_{18}-StageTips (2).

Analysis by nanoLC-HDMS^E

Mass spectrometry was performed on a Synapt G2-S mass spectrometer (Waters, Manchester, UK) using Masslynx version 4.1 coupled to a nanoAcquity UPLC system

(Waters) via a NanoLockSpray ion source using a PicoTip Emitter (New Objective, Woburn, MA, USA). Mobile phase A contained 0.1% formic acid in water, and mobile phase B contained 0.1% formic acid in acetonitrile. Three analytical replicates per sample were injected. Peptide samples corresponding to approximately 150 ng of digested protein supplemented with 20 fmol of Hi3 Phos B standard for protein absolute quantification (Waters) were trapped and desalted using a precolumn (nanoAcquity UPLC Symmetry C18, 5 µm, 180 µm x 20 mm, Waters) at a flow rate of 10 µl/min for 4 min with 99.9% A. Peptides were separated on an analytical column (ACQUITY UPLC HSS T3, 1.8 µm, 75 µm x 200 mm, Waters) at a flow rate of 300 nl/min using a gradient from 3% to 35% B over 90 min. After separation, the column was washed with 85% B for 10 min and re-equilibrated with 97% A for 20 min. The column temperature was maintained at 35 °C. As a reference compound, 100 fmol/µl [Glu^{1}]-Fibrinopeptide B was delivered at 500 nl/min to the reference sprayer of the NanoLockSpray source.

The SYNAPT G2-S instrument was operated in data-independent mode (3), characterized by parallel fragmentation of multiple precursor ions in combination with ion-mobility separation as an additional dimension of separation (referred to as $HDMS^{E}$) (4). By executing alternate scans at low and elevated collision energy (CE) of each 0.5 sec, information on precursor and fragment ions, respectively, was acquired. In low-energy MS mode acquisitions were performed at constant CE of 4 eV whereas drift time-dependent CE settings were applied in elevated-energy MS mode. The values of drift time-dependent CE settings were used as described (5). Lock spray was acquired once every 30 s for a 1 s period.

nanoLC-HDMS^E data processing, protein identification and quantification

Progenesis QI for Proteomics version 2.0 (Nonlinear Dynamics, Newcastle upon Tyne, UK) (6) was used for raw data processing, protein identification and label free quantification. For each biological experiment the raw data from the three analytical replicates of WT, Δ*marS*, and Δ*marS*::*marS* samples from the same growth phase were imported into a separate Progenesis experiment.

During import, data were lock mass-corrected using the doubly charged monoisotopic ion of [Glu^1]-Fibrinopeptide B. For Apex3D processing, the thresholds for low and high energy scan ions and total ion intensity were set to 135, 30, and 750 counts, respectively. Following alignment to compensate for between-run variation in the LC separation, singly charged ions were filtered out. Peak picking parameters included (i) sensitivity set automatic, (ii) minimum chromatographic peak width of 0.15 min, and (iii) maximum ion charge of +4. The "Between-subject design" was used to group the three analytical replicates of each sample into one group.

For the database search a database containing 1701 protein sequences from *Streptococcus pyogenes* serotype M49 (strain NZ131) (UniProt release 2015_01) appended with the sequences of rabbit phosphorylase B (P00489) and porcine trypsin was compiled. Precursor and fragment ion mass tolerances were automatically determined. One missing cleavage site was allowed, oxidation of methionine residues was considered as variable modification, and carbamidomethylation of cysteines as fixed modification. The false discovery rate (FDR) based on the search of a reversed database was set to 1%. Peptides were required to be identified by at least three fragment ions and proteins by at least seven fragment ions and two peptides. With the subsequent filtering steps peptides were removed that had (i) a score below 4.5, (ii) less than 2 hits; i.e. peptides which were identified only once within the nine LC-HDMS^E runs, (iii) a mass error above 7.0 ppm,

(iv) an Anova p-value above -0.05, (v) been identified by charge state deconvolution only. Proteins were quantified by the absolute quantification Hi3 method using Hi3 Phos B Standard (Waters) as reference (7). The coefficient of variation (CV) among the three analytical replicates per sample was estimated as the ratio of the standard deviation to the mean multiplied by 100. The overall CV for the quantifications of all proteins was 4.2, 4.8, and 4.1 for the WT, Δ*marS*, and Δ*marS*::*marS* samples, respectively. For the differentially expressed proteins reported in Table 2 the mean CV was 4.6, 9.5, and 4.3 for the WT, Δ*marS*, and Δ*marS*::*marS* samples, respectively, indicating an increased variation for differentially expressed proteins in Δ*marS*. This was related to a significantly reduced protein amount of these proteins in Δ*marS*. Protein abundance differences were quantified as fold change by calculating the ratio of WT to Δ*marS* values and WT to Δ*marS*::*marS* values, respectively. The fold change was first calculated from the mean protein abundances of the analytical replicates per sample and subsequently the mean of the fold change values from the biological replications was computed.

1. **Masuda T, Tomita M, Ishihama Y**. 2008. Phase transfer surfactant-aided trypsin digestion for membrane proteome analysis. J Proteome Res **7**:731-740. doi:10.1021/pr700658q [doi].

2. **Rappsilber J, Mann M, Ishihama Y**. 2007. Protocol for micro-purification, enrichment, pre-fractionation and storage of peptides for proteomics using StageTips. Nat Protoc **2**:1896-1906. doi:nprot.2007.261 [pii];10.1038/nprot.2007.261 [doi].

3. **Geromanos SJ, Vissers JP, Silva JC, Dorschel CA, Li GZ, Gorenstein MV, Bateman RH, Langridge JI**. 2009. The detection, correlation, and comparison of peptide precursor and product ions from data independent LC-MS with data dependant LC-MS/MS. Proteomics **9**:1683-1695. doi:10.1002/pmic.200800562 [doi].

4. **Shliaha PV, Bond NJ, Gatto L, Lilley KS**. 2013. Effects of traveling wave ion mobility separation on data independent acquisition in proteomics studies. J Proteome Res **12**:2323-2339. doi:10.1021/pr300775k [doi].

5. **Distler U, Kuharev J, Navarro P, Levin Y, Schild H, Tenzer S**. 2014. Drift time-specific collision energies enable deep-coverage data-independent acquisition proteomics. Nat Methods **11**:167-170. doi:nmeth.2767 [pii];10.1038/nmeth.2767 [doi].

6. **Kuharev J, Navarro P, Distler U, Jahn O, Tenzer S**. 2014. In-depth evaluation of software tools for data-independent acquisition based label-free quantification. Proteomics. doi:10.1002/pmic.201400396 [doi].

7. **Silva JC, Gorenstein MV, Li GZ, Vissers JP, Geromanos SJ**. 2006. Absolute quantification of proteins by LCMSE: a virtue of parallel MS acquisition. Mol Cell Proteomics **5**:144-156. doi:M500230-MCP200 [pii];10.1074/mcp.M500230-MCP200 [doi].

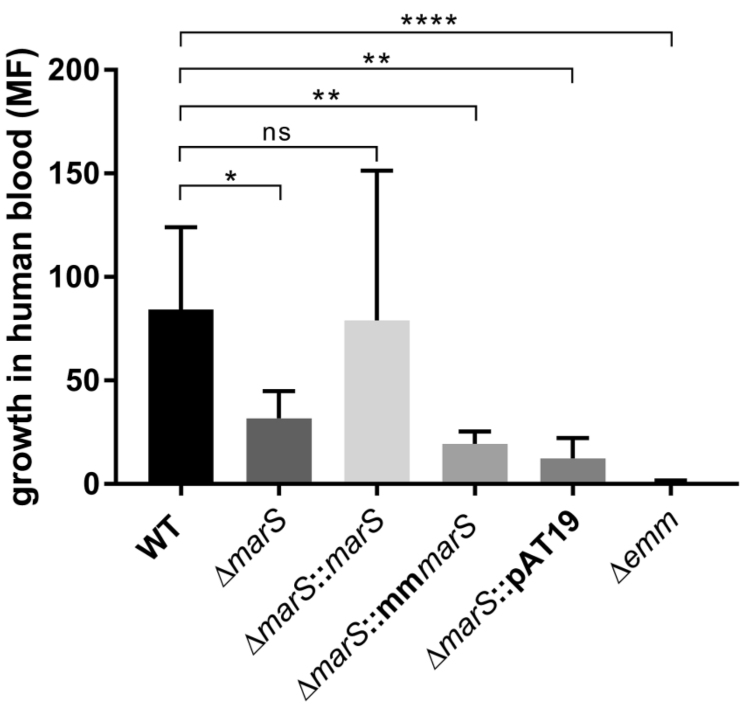

Supplementary Figure S1. Bacterial growth in human blood. Growth in human blood of Δ*marS* and Δ*marS::marS* in comparison to WT, n=12, and growth in human blood of the mismatch complementation strain, in which two bases situated in the putative binding site have been exchanged (88-CC-89/88-GG-89), mmΔ*marS::marS*, an empty vector control, Δ*marS::*pAT19, and an *emm* deletion strain, Δ*emm*, in comparison to WT, n=6. Data are presented as MF (mean values ± standard deviation). Statistical significance was determined using the Kruskal-Wallis test (using Dunn's correction for multiple comparisons). Differences between samples were expressed as "ns = not significant" (P ≥ 0.05), marginally significant (P < 0.05)*, significant (P < 0.01)**, and highly significant (P < 0.001)****.

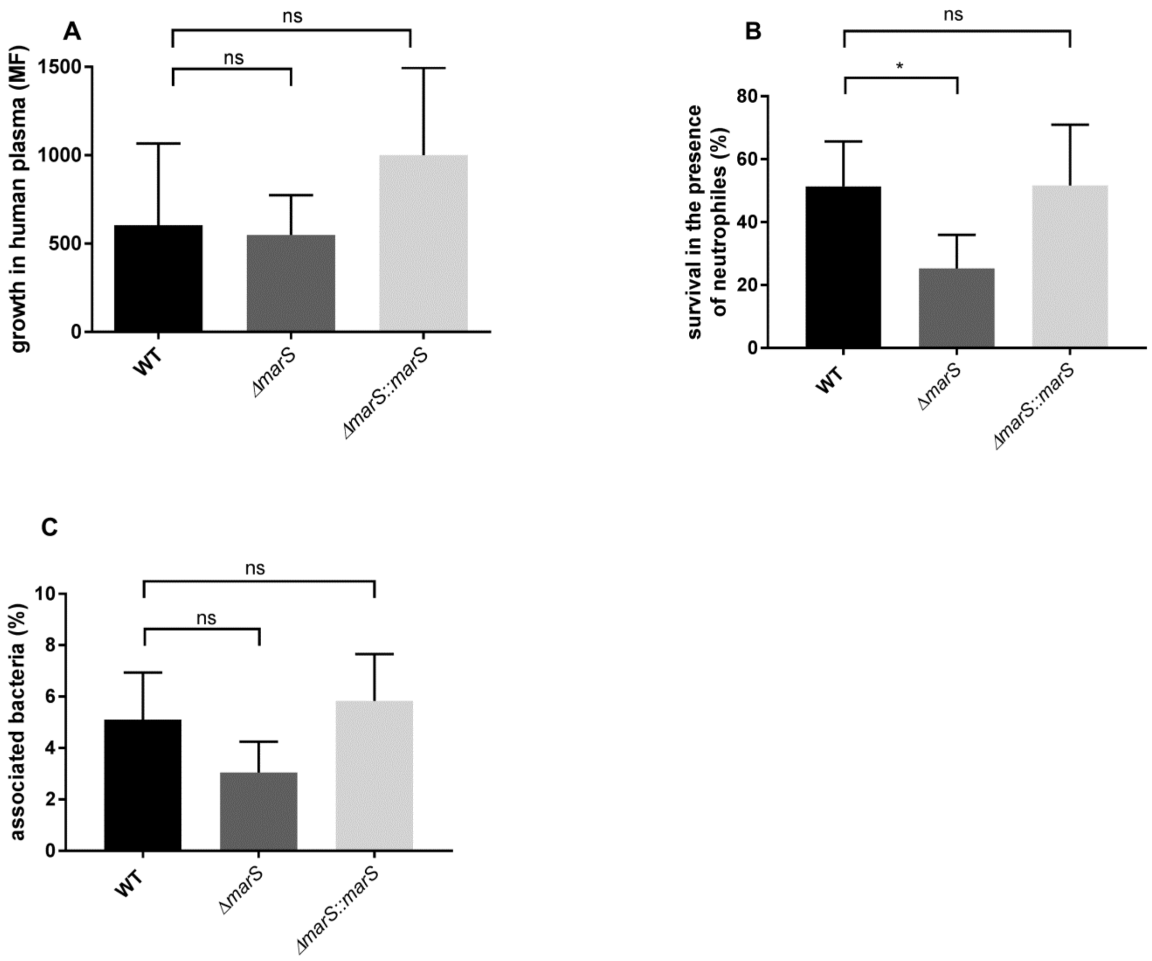

Supplementary Figure S2. Bacterial growth in human plasma and survival in the presence of neutrophiles. (A) Growth in human plasma of Δ*marS* and Δ*marS::marS* in comparison to WT, n=4, Data are presented as MF (mean values ± standard deviation). (B) Survival of Δ*marS* and Δ*marS*::*marS* in comparison to WT after incubation for 30 min with human neutrophils, n=5. Data are presented as % survival (mean values ± standard deviation). (C) Abundance of associated Δ*marS* and Δ*marS*::*marS* bacteria in comparison to WT after incubation with neutrophils n=5. The Data are presented as % associated bacteria (mean values ± standard deviation). Statistical significance was determined using the Kruskal-Wallis test (using Dunn’s correction for multiple comparisons). Differences between samples were expressed as “ns = not significant” ($P \geq 0.05$) and marginally significant ($P < 0.05$)*.

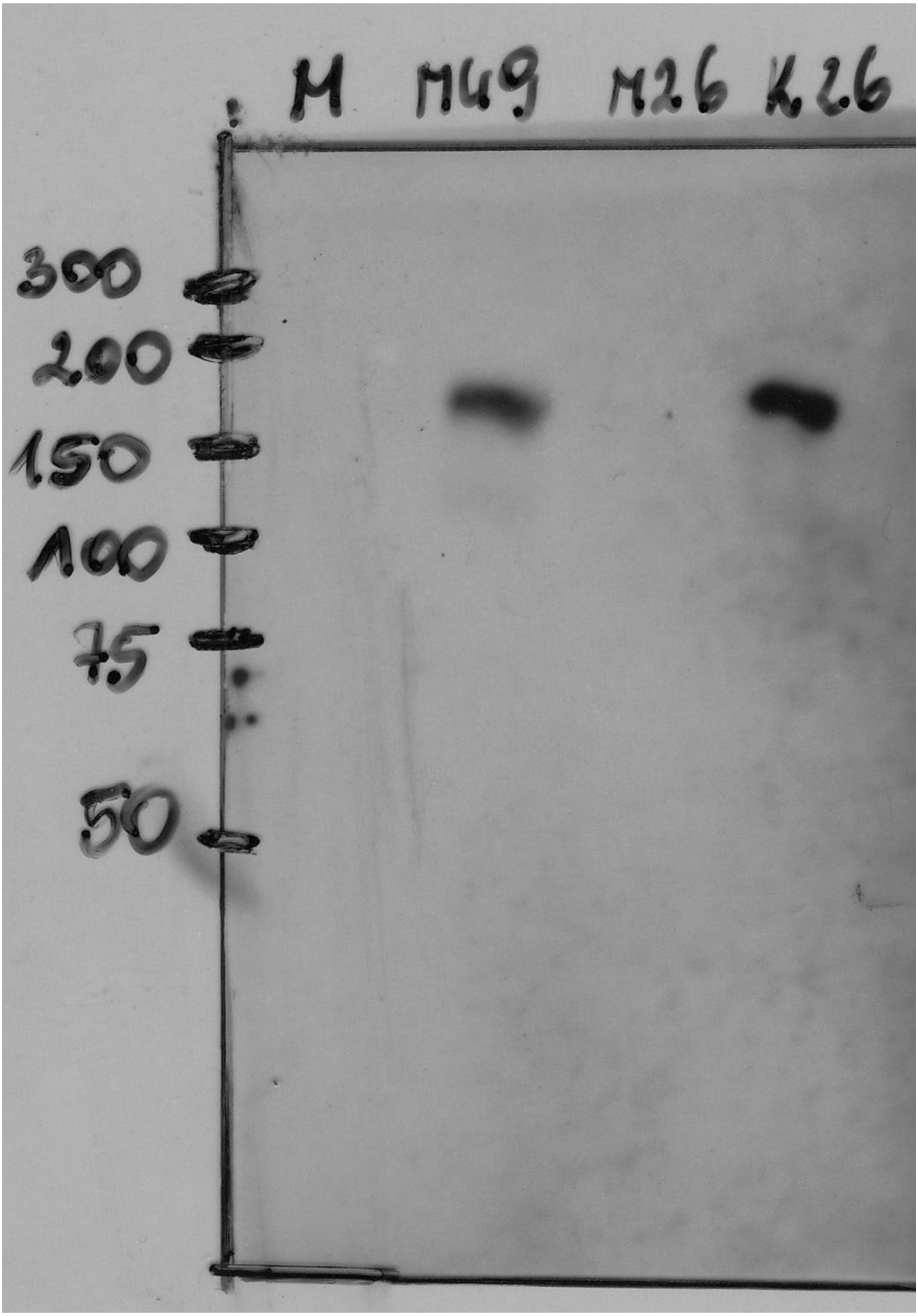

Supplementary Figure S3. Original Northern blot X-ray film scan. Northern blot analyses of marS expression during growth in THY medium (Fig 1C).
